# Quasistatic tensile and flexural behaviors of fiber metal laminates after subjecting to uniaxial tensile impact loading

**DOI:** 10.1038/s41598-025-99159-6

**Published:** 2025-05-08

**Authors:** Islam El-Sagheer, Amr A. Abd-Elhady, Hossam El-Din M. Sallam

**Affiliations:** 1 Mechanical Design Department, Faculty of Engineering, Helwan University, Cairo, 11718 Egypt; 2 Materials Engineering Department, Zagazig University, Zagazig, 44519 Egypt

**Keywords:** Fiber-metal laminate (FML), Progressive damage, tensile test, Flexural test, Tensile-impact test, After impact test, Composites, Mechanical engineering

## Abstract

This study represents a novel contribution regarding the behavior of fiber-metal laminates (FMLs) under tensile impact loading. The experimental program included axial tensile-impact tests and quasi-static tensile and flexural tests after impact. The FMLs’ core consisted of an epoxy matrix reinforced with long glass fibers, and their skins had 1050 aluminum plates. Several variables were investigated, including the lay-up methods and the insertion of the [90°] layers within [Al/0°/Al] and [Al/0°/0°/Al] FML specimens. The results showed that the residual tensile load of [Al/0°/90°/0°/Al] sandwich specimens’ lay-up method was 6.65 *kN*, from the original ultimate load of 17.69 *kN*. For bending after impact, the residual load was 0.79 *kN*, from the original one of 1.46 *kN*. Although inserting the [90°] layers provided low tensile strength, it effectively blunted the propagating crack in the load’s direction under either the quasi-static tensile or the tensile-impact tests. For progressive damage, the [0°] layer showed fiber breakage and fiber-kinking, and the [90°] layer exhibited critical matrix cracking. Meanwhile, the aluminum plates exhibited a transverse crack during the delamination between the composite core and aluminum plates. During the tensile-impact test, two divisions of delamination were observed: partial delamination aside from [0°] and severe delamination with [90°].

**Supplementary Information:**

The online version contains supplementary material available at 10.1038/s41598-025-99159-6.

## Introduction

Fiber-metal laminates (FMLs) and fiber-reinforced polymeric (FRP) composites have garnered significant interest in the past thirty years because of their superior strength-to-weight. This has led to their application in heavy industries, including automotive and marine construction. Metal (plates) and polymeric material (core) are used to create FMLs. According to earlier research, one of the most often used metals in these structures is aluminum plates^1,2^. Looking at the history of FMLs, these sandwich materials were primarily developed to address the poor fatigue resistance of metal alloys. On the other hand, tensile^1,2,3,4,5,6,7,8,9,10^, flexural^11,12,13,14,15,16^, and post-impact loadings^17,18,19,20,21,22,23,24,25,26,27^ are just a few of the loading situations that FMLs and FRPs are exposed to.

Because it links the fiber orientation to the principal stress applied to the FMLs, the tensile strength of [0°/90°] of the FMLs was greater than that of [-45°/+45°]^3^. Comparing FMLs with two and three layers of kenaf fabric, the maximum tensile characteristics were obtained by incorporating a single layer of kenaf fabric into untreated FMLs. FMLs with a fiber orientation of [0°/90°] exhibited superior tensile characteristics than those with a fiber orientation of ± 45°^4^. The introduction of jute fibers between basalt fibers showed the highest tensile strength and modulus of elasticity values in 2024-T6 aluminum sandwiches^5^. Furthermore, a split Hopkinson tensile bar approach was used to examine the influence of strain rate on the tensile behavior of FMLs based on aluminum and GFRP. Fiber bridging and the extra strain gained in aluminum gave the FMLs a tensile strength about 5% higher than GFRP^6,7^. The failure sequence was visible as the metal layers were plastically distorted along with the elastically distorted fiber layers, aside from complete delamination^8,9,10^.

Adding graphene nanoplatelets (GNPs) to composite laminates improved the flexural characteristics of FMLs after being submerged in the 3.5 wt% NaCl solution^12^. Alternatively, increasing the duration of immersion decreased the flexural characteristics of specimens. The mechanical characteristics of the orthogonal 45° and 90° laminates were analogous, and the fiber direction did not influence the flexural characteristics of CFRP/Al laminates in the elastic phase. However, CFRP/Al laminates were influenced only by the layers in 0° direction^13^. The bending failure phases of Glass Laminate Aluminum Reinforced Epoxy (GLARE) laminate are the elastic, plastic, delamination, and fiber breakage^13,14,15,16^.

The post-impact flexural test showed that hybrid carbon fiber and aramid fiber fabric laminates exhibited significant brittle damage characteristics as the flexural strength decreased by 13.9% after impact^17^. Adding GNPs and silica nanoparticles exhibited the best peak load and compression-after-impact (CAI) strength. This resulted from the increased damage suppression, which was seen in impact trials conducted at low velocities^18,19^. Likewise, hybrid laminates showed a comparatively smaller damage area and greater CAI strength than woven carbon laminates, which depicted a 26% lower peak force value^20^. Furthermore, a global buckling in the stiffeners, cracks in the transition regions, and delamination in the skin and stiffener were all visible in the CAI behaviors of rib-stiffened FRP panels^21^. The GLARE was also evaluated for impact and post-impact damage propagation under flexure. Their results showed a 15% reduction in flexural strength compared to the non-impacted specimen. Furthermore, pre-existing damage accelerated the rate of damage development in the impacted specimen’s fibers and matrix^22^. Hybrid carbon fiber and aramid fabric laminates also showed significant brittle degradation features, with the lowest fracture strength occurring after impact^23^. The application of intra-ply hybrid patches decreased the impact absorption of energy by 10.17% compared to the virgin samples^24^.

All the previous investigations on the behavior of FMLs under impact loading mainly focused on penetration or puncture-impact tests^25,26,27^. Nevertheless, studies on tensile-impact, flexural-impact, or compression-impact tests are notably absent. To the best of the authors’ knowledge, the tensile-impact test represents a novel approach to impact loading for FMLs, and consequently, there is a lack of research on tensile and flexural behaviors after the tensile-impact test. This observation is supported by the fact that most studies focus on the post-impact behavior of the composite laminates^18–21,28−30^, whereas the post-impact behavior of FMLs has received limited attention^22,31^. In reality, several members/components could be subjected to axial impact load. For example, aircraft on aircraft carriers are subjected to severe tensile impact loads, which affect their tail hooks during landing. Another example is when tension members in a truss girder are subjected to dynamic or impact loads. Furthermore, no previous studies have investigated the effect of FML manufacturing procedures on their structural and failure behavior^1,2,3,4,5,6,7,8,9,10,11,12,13,14,15,16^. The most common manufacturing techniques are the sandwich lay-up technique (the skin plates are bonded to the core after curing) and the hand lay-up technique (the skin plates are bonded to the core during fabrication). Therefore, one of the main objectives of the present work is to fill this gap in the literature. Another parameter worth examining in the present manuscript is inserting the [90°] layer in the core of FMLs subjected to axial loading. Inserting the [90°] layer may decrease the tensile strength of FMLs due to the increase in the cross-sectional area with decreased stiffness of such a layer. However, its insertion may increase the absorbed energy of FMLs due to blunting the front of the propagating crack. Another contribution of the present study is investigating the residual tensile and flexural strength of FMLs after the tensile-impact test. These loading conditions are relevant to real-world applications, especially in automotive systems. By studying such factors in the present work, their results may lead to improving the performance of FML and ensuring the long-term reliability of the structure.

## Experimental work

in the present study, a novel contribution regarding the behavior of FMLs under tensile impact loading and the residual tensile properties after tensile impact loading. the experimental program included axial tensile-impact tests and **quasi-**static tensile and flexural tests after impact to achieve this novelty. the FMLs’ core consisted of an epoxy matrix reinforced with long glass fibers, and their skins had 1050 aluminum plates.

### Materials

The 1050 aluminum plates of 0.5 *mm* thickness were treated as mentioned^32^: first, they were lightly abrasive with #400 grit sandpaper. Next, they were acid-washed with HCl containing 11% volume to increase the surface roughness at room temperature for 30 min. Lastly, they were washed with distilled water and allowed to dry. Finally, using an alkaline medium, they were submerged in a 5% NaOH solvent for 5 min at 70 °C. The laminated composite was made from unidirectional glass fibers (manufactured by Jushi, Zhejiang, China), which were infused with epoxy (Kemapoxy 150, provided by CMB, Giza, Egypt) with a resin-to-hardener ratio of 2:1, as the pot life is 30-minute. According to the CMB manufacturer, it is also a solvent-free, non-pigmented liquid resin that hardens completely after seven days. Conventional fiber-reinforced polymer (FRP) was made using a hand lay-up approach. It is worth noting that the average thickness of one ply was 0.833 mm. The physical and mechanical properties of aluminum, epoxy, and fiberglass are listed in Tables 1 and 2.

**Table 1:** Fiberglass, epoxy, and aluminum physical properties.

Material	Physical properties	Value
E-glass fiber	Filament diameter	13 μm
	Linear density	2400 (tex)
Epoxy	Density	1.11 ± 0.02 kg/L
	Abrasion resistance	1–6 cm^3^/50 cm^2^
	Temperature resistance	Humid 90 °C/dry 140 °C
Aluminum	Density	2700 (kg/m^3^)

**Table 2:** Fiberglass, epoxy, and aluminum mechanical properties.

Mechanical properties	E-glass fiber	Epoxy	Aluminum
Modulus of elasticity, *E*, (GPa)	82	3.24	70
In-plane shear modulus, *G*, (GPa)	30.13	1.19	26.32
Poisson’s ratio, *ν*	0.22	0.36	0.3

### Specimen Preparation

**Fig. 1:**
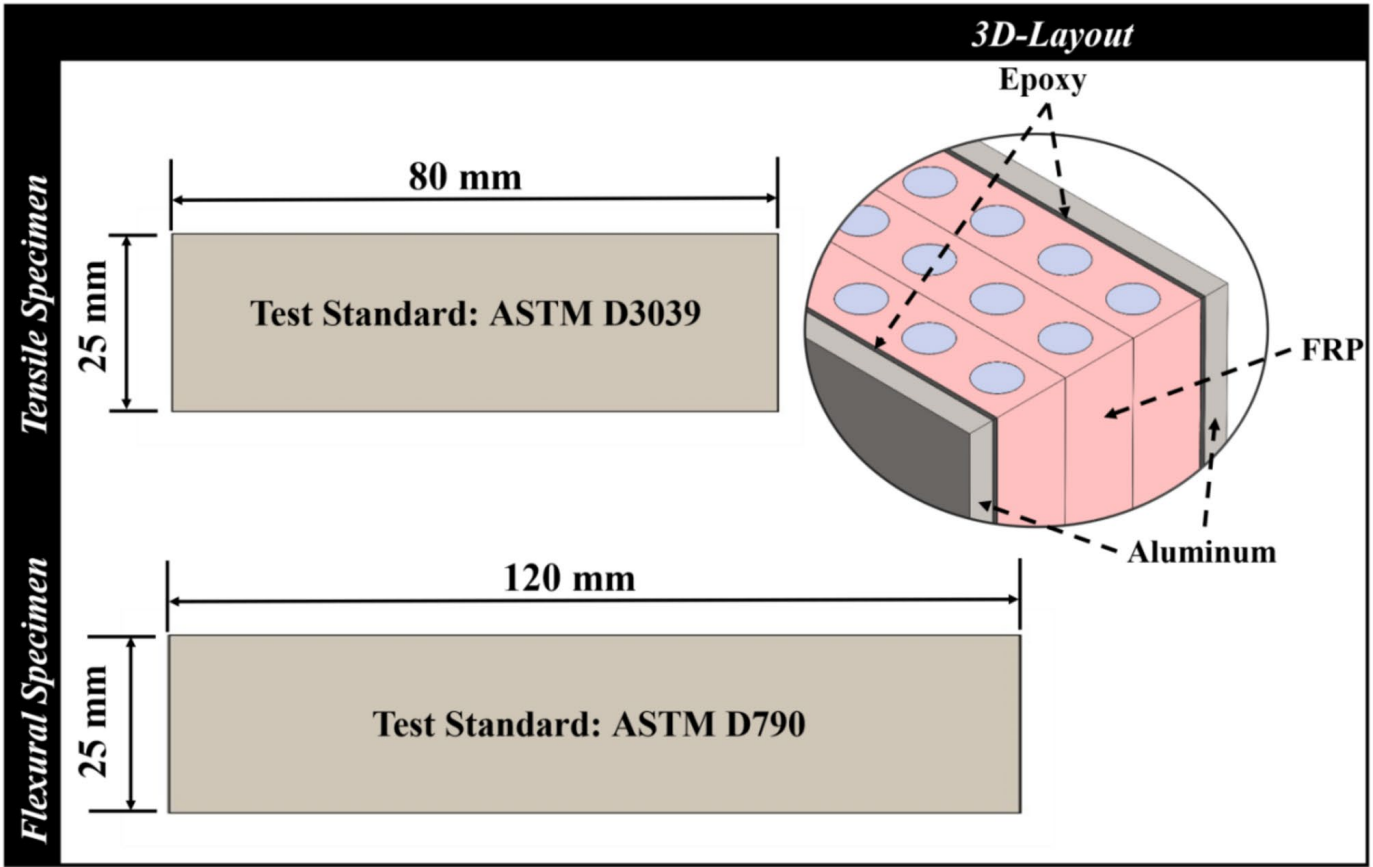
The specimen layout.

Using a hand lay-up technique, the FML plates were first manufactured with an average thickness of 3.5 *mm*. Then, the plates were trimmed into various specimen sizes using an electric saw. It is worth noting that the saw was utilized at a very high speed to minimize the potential defects. As a result, they were fitted for tensile and flexural testing; the specimen sizes are shown in Fig. 1. The specimens are divided into two main groups to study the following two parameters:

**Group 1: Studying the influence of lay-up techniques**.

a- FML-sandwich technique of [Al/0°/90°/0°/Al].

b- FML-hand lay-up technique of [Al/0°/90°/0°/Al].

c- FRP-hand lay-up technique of [0°/90°/0°].

**Group 2: Studying the influence of inserting [90**°**] layers inside FML**.

a- [Al/0°/Al] vs.[Al/0°/Al] with outer layers of [90°] (to become [Al/90°/0°/90°/Al]).

b-[Al/0°/0°/Al] vs. [Al/0°/0°/Al] with intermediate layer of [90°] (to become [Al/0°/90°/0°/Al]).

It is worth noting that the average fiber volume fractions were determined by ASTM D3171^33^, and its value (***v***_***f***_ = 39%). Moreover, when the aluminum plates and fiber/epoxy composite are laid up simultaneously,

Specifications of the composite samples, including composite core density, thickness, and the average fiber volume fractions determined by ASTM D3171^33^, are listed in Table 3. The rationale behind using the FML-sandwich lay-up technique is that some industries (for mass production) used to increase the time by early preparation of FRP sheets, as prepreg (a pre-impregnated composite material with a resin), then the metal plates are added in the next phase. However, the FML-hand lay-up technique is used for specific industries or sophisticated shapes to build the metal skins and FRP lay-up together to fit the particular product or shape. In the case of the FML-sandwich lay-up technique, the bond between the FML’s core and skins may be excellent but not perfect; hence, the possibility of debonding occurs, and subsequently, energy consumption occurs. However, the FML-hand lay-up technique has an ideal bond without the possibility of debonding between the core and the skin; hence, this technique may lose this source/advantage of energy consumption. According to the present study, it is a hand lay-up technique when the aluminum plates and fiber/epoxy composite are laid up simultaneously. The fiber/epoxy composite is laid up to reach curing first, then the aluminum plates are placed, which is a sandwich lay-up technique.

### Testing configuration

**Fig. 2:**
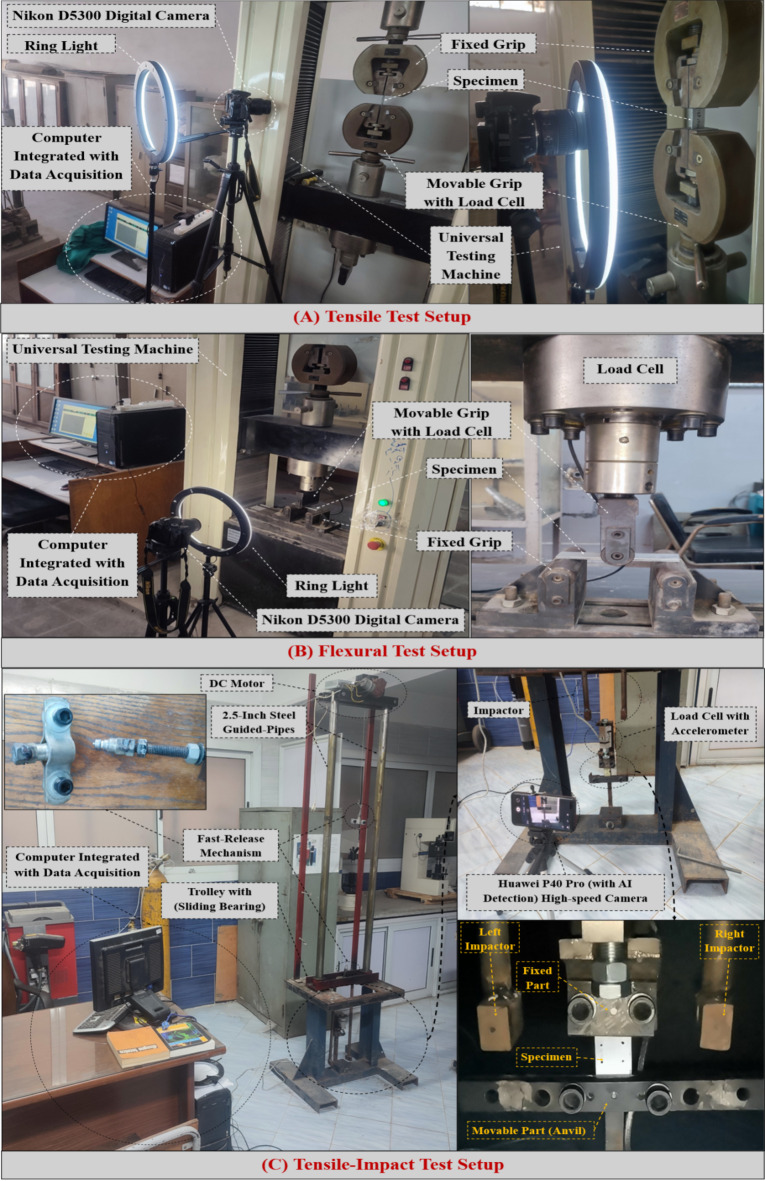
(**A**) Tensile test setup, (**B**) Flexural test setup, and (**C**) Tensile-Impact test setup.

The tests aim to determine how much the tensile-impact absorbed energy and the strengths of the flexural and tensile without impact and after impact of the FMLs, which are affected by the parameters mentioned above. Tensile and tensile after impact (TAI) testing involved firmly clamping each specimen into the universal testing apparatus and aligning it along its longitudinal axis, as shown in Fig. 2(A). The specimens were loaded parallel to their axial. In displacement control, the tensile and TAI tests were conducted at an average strain rate of 2 mm/min. as per ASTM D3039^34^. A three-point bending test was adopted to measure the flexural and flexural after-impact (FAI) behavior, as shown in Fig. 2(B). In the case of the flexural and FAI tests, the specimens were loaded at an average speed of 5 *mm* per minute until they achieved their final failure, according to ASTM D3039^35^. A digital camera (Nikon D5300) was used for the tensile and TAI tests as well as the flexural and FAI tests. Tensile and flexural strengths were examined after load-displacement curves were obtained for each specimen.

**Table 3:** The sample specifications.

Material	Composite core density (kg/m^3^)	Thickness (mm)	Fiber volume fraction (v_f_%)
[0°/90°/0°]	1457.7	2.5	39
[Al/0°/Al]		1.833	
[Al/0°/0°/Al]		2.67	
[Al/0°/90°/0°/Al]		3.5	
[Al/90°/0°/90°/Al]		3.5	
[Al]-single layer		0.5	

Figure 2C shows the hand-made axial tensile impact testing machine using the drop-weight technique. The machine was designed and manufactured at the Mechanical Design Department, Helwan University, Cairo, Egypt. The main frame was made from steel (St. 42), and the guided bars were 2.5-inch steel pipes. The trolley carried two impactors (with flat-rectangle ends), a DC motor adjusted the trolley’s level, and the trolley slid through a guide by a lubricated slider bearing (made from Teflon). A fast-release mechanism through steel wire was used to release the impactor. The weight of the impactor could reach up to 10 *kg*, while the clear distance between the impactor and the anvil (moving part in Fig. 2(C) could reach up to 1.2 *m*.

The specimen was positioned in an upper-fixed portion (connected to a load cell that was coupled with the accelerometer ADXL345), while the bottom of the specimen was placed in conjunction with a moveable part (anvil), which was stroked by the impactor. In the present work, the weight of the impactor was 1.05 *kg*, and the clear height was 0.3 *m*, i.e., the impact energy of 3*J*. A high-speed camera (Huawei P40 Pro with AI detection) based on ISO 8256^36^ for the tensile-impact test, the displacement was measured according to the imaging process between two points in the sample surface (the first point at the fixed grip and the second point near the movable grip). During the tests, the force and time were documented. From the displacement-time curve and the force-time curve, the force-displacement curve can be easily constructed. Subsequently, the absorbed energy was calculated from the area under the force-displacement curve. MATLAB was employed to calculate the area under the curve using an implemented integration function. Note that five samples are used for each test case.

## Results and discussion

All results are listed in Tables 4, 5, 6, 7, 8, 9, 10, 11, 12 and 13. The Supplementary Information (Table S1 and Note N1) summarizes the statistical (repeatability) analysis for all current measurements. The results presented in Table S1 showed that the coefficient of variation (COV) ranged from 0.36 to 18.65%, indicating a higher accuracy of results. A “relative damage” term was suggested to measure the tensile impact loading damage in FML specimens. This term was calculated by subtracting the value of such variable measured from a specimen subjected to impact load from the corresponding one measured from the virgin specimen (impact-free), all divided by the value measured from the virgin specimen. It is worth noting that the value of this term may be negative (decrement) or positive (increment).

### Group 1: effect of lay-up technique

#### Axial tensile-impact test results

**Fig. 3:**
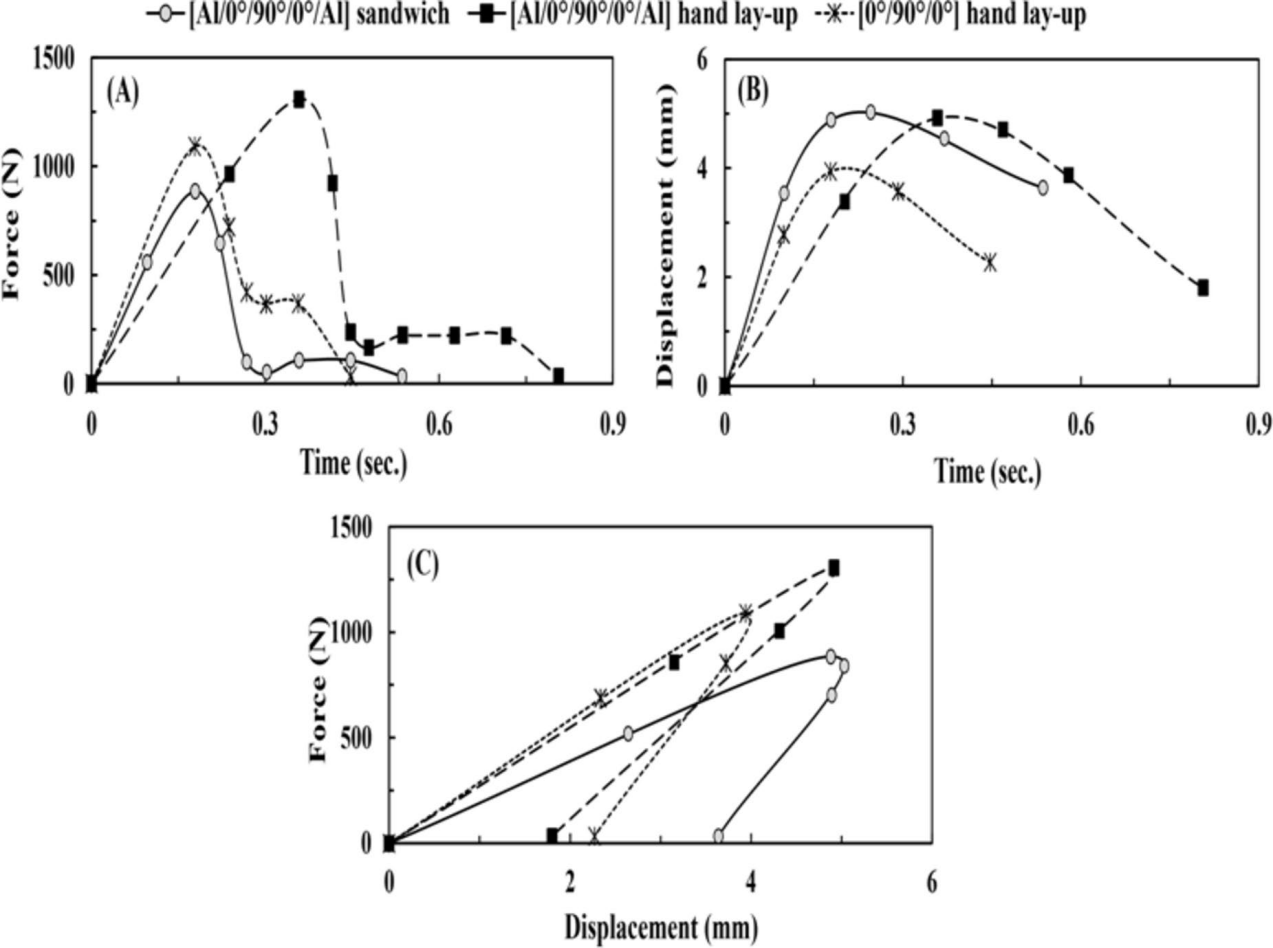
(**A**) Force vs. time (through tensile-impact) for group 1, (**B**) displacement vs. time (through tensile-impact) for group 1, and (**C**) force vs. displacement (through tensile-impact) for group 1.

Table 4; Fig. 3 demonstrate that the [Al/0°/90°/0°/Al] sandwich specimen exhibited the greatest absorbed energy compared to [0°/90°/0°] and [Al/0°/90°/0°/Al] hand lay-up specimens. Conversely, it showed the least value of the peak tensile-impact load as it replaced its energy absorption domain from load-field to displacement-field, illustrating a larger permanent extension of 3.64 mm. However, it absorbs the greatest impact energy because it possesses the three most efficient mediums for impact energy absorption. The composite core of the fiber-reinforced/epoxy, the aluminum plates that transform energy absorption into plasticity, and the debonding between the aluminum plates and the composite (fiber-reinforced/epoxy). The debonding occurred between them due to the quality of the bond in such a technique, which is excellent but not perfect. The interlaminar bonding is the only visible difference between the [Al/0°/90°/0°/Al] sandwich specimen and the [Al/0°/90°/0°/Al] hand lay-up specimen. The FMLs’ samples show partial delamination between the FRP core and the aluminum plates, while the FRP’s sample illustrates no delamination. There is no evidence of crack existence or propagation, as recorded by Zhang et al.^37^; below the impact energy of 20 *J*, there seemed to be no crack.

#### Quasi-static tensile test results

**Fig. 4:**
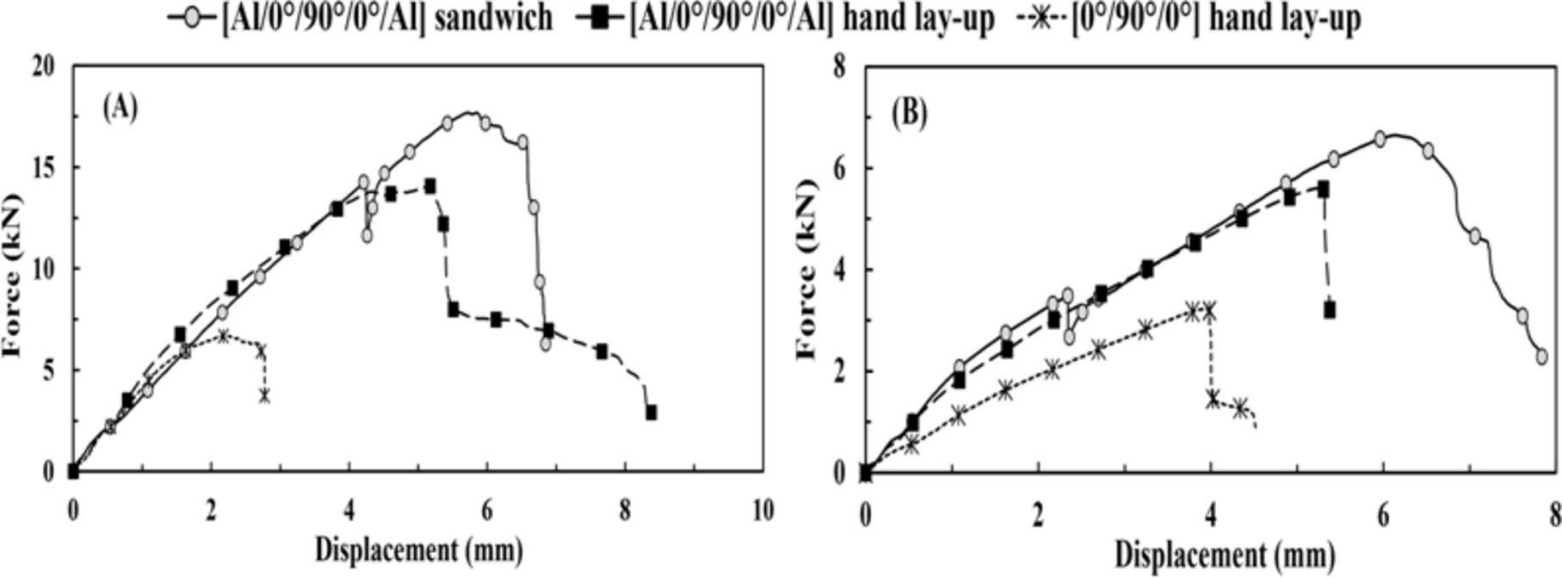
(**A**) Force vs. displacement (without tensile-impact) under tensile test for group 1 and (**B**) force vs. displacement (after tensile-impact) under tensile test for group 1.

The two techniques of FMLs’ specimen manufacturing, i.e., sandwich and hand lay-up, with the same pattern and fiber volume fraction of core [0°/90°/0°], are compared concerning the resistance of axial tensile load without and after impact. It was found that the specimens made using both techniques had the same stiffness. However, the [Al/0°/90°/0°/Al] sandwich specimen had the strongest resistance to external force for virgin and TAI specimens, as shown in Table 5; Fig. 4. Even with the decrease in peak load and tensile stiffness values of 62.41 and 64.92%, respectively, after the TAI test, as shown in Fig. 4B; Table 6.

**Table 4:** The experimental results based on the effect of the lay-up technique under the tensile-impact test.

Specimen	Absorbed energy, (J)	Avg. peak load, (kN)	Permanent extension, (mm)	Standard deviation (σ), (J)	Coefficient of variation (COV%)
[Al/0°/90°/0°/Al] hand lay-up	1.13	1.3	1.8	0.06	4.87
[Al/0°/90°/0°/Al] sandwich	1.6	0.89	3.64	0.17	18.65
[0°/90°/0°] hand lay-up	1.21	1.09	2.27	0.03	2.9

The reason why the [Al/0°/90°/0°/Al] sandwich had enhanced resistance more than the [Al/0°/90°/0°/Al] hand lay-up specimen is that the sandwich technique provides a good bond between the aluminum and the core, absorbing energy into interlaminar damage. However, the hand lay-up technique presented a perfect bond between aluminum and the core, in which FMLs behaved as monolithic material. Both methods showed delamination between the core and the FMLs’ skin (at the final failure), while the sandwich technique showed a much wider scale of delamination than the hand lay-up technique. In contrast, when the aluminum plates were removed, [0°/90°/0°] hand lay-up specimen showed decreased tensile resistance and ductility, which demonstrated the highest reduction in the tensile stiffness and increase in deformation (corresponding to peak load) values of 72.59% and 75.89%, respectively. However, it showed the least reduction in the peak load value of 51.79%. This indicated that the fibers are roughly enough to endure most of the load, unlike the matrix exposed to intralaminar damage (matrix cracking), as it records the maximum increase in total deformation. There is no delamination between core/FRP layers, as the delamination requires more inter-damage energy (between FRP layers) than intra-damage energy (for matrix cracking).

**Table 5:** The effect of the lay-up technique on the results of the tensile test.

Specimen	Avg. peak load (kN)		Coefficient of variation (COV%)		Displacement corresponding to peak load (mm)		Max. displacement (mm)	
	Without impact	After impact	Without impact	After impact	Without impact	After impact	Without impact	After impact
[Al/0°/90°/0°/Al] hand lay-up	14.02	5.59	8.23	4.27	5.13	5.24	8.33	5.39
[Al/0°/90°/0°/Al] sandwich	17.69	6.65	3.62	2.73	5.73	6.14	6.84	7.82
[0°/90°/0°] hand lay-up	6.68	3.22	3.79	6.87	2.24	3.94	2.78	4.52

**Table 6:** The effect of the lay-up technique on the relative damage measured through the tensile test.

Specimen	Relative peak load (%)	Relative displacement corresponding to peak load (%)	Relative max. displacement (%)	Relative tensile stiffness (%)
[Al/0°/90°/0°/Al] hand lay-up	− 60.13	2.14	− 35.3	− 60.96
[Al/0°/90°/0°/Al] sandwich	− 62.41	7.16	14.33	− 64.92
[0°/90°/0°] hand lay-up	− 51.79	75.89	62.59	− 72.59

#### Quasi-static flexural test results

**Fig. 5:**
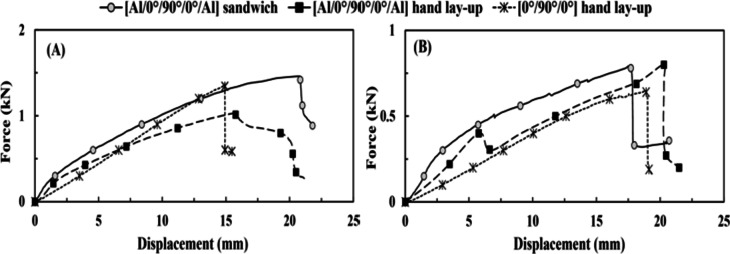
(**A**) Force vs. displacement (without tensile-impact) under flexure test for group 1 and (**B**) force vs. displacement (after tensile-impact) under flexure test for group 1.

In the case of the flexural test, the [Al/0°/90°/0°/Al] sandwich specimen depicted higher resistance with the ultimate deflection of 21.8 *mm*, as shown in Table 7; Fig. 5(A), more than [Al/0°/90°/0°/Al] hand lay-up specimen. Furthermore, the [Al/0°/90°/0°/Al] hand lay-up specimen had resistance to external force with the smallest peak load decrease of 22.33%, which reflected as the greatest increase in deflection (corresponding to peak load) by 30.65%, i.e., a slightly higher than the [Al/0°/90°/0°/Al] sandwich specimen but a higher than the [0°/90°/0°] hand lay-up specimen, as shown in Fig. 5(B) and Table 8.

**Table 7:** The effect of the lay-up technique on the results of the flexural test.

Specimen	Avg. peak load (kN)		Coefficient of variation (COV%)		Deflection corresponding to peak load (mm)		Max. deflection (mm)	
	Without impact	After impact	Without impact	After impact	Without impact	After impact	Without impact	After impact
[Al/0°/90°/0°/Al] hand lay-up	1.03	0.8	5.37	3.95	15.53	20.29	21.15	21.5
[Al/0°/90°/0°/Al] sandwich	1.46	0.79	17.87	12.01	20.81	17.68	21.8	20.65
[0°/90°/0°] hand lay-up	1.36	0.65	8.14	6.08	14.89	18.85	15.34	19.33

According to the FAI test, the [Al/0°/90°/0°/Al] hand lay-up specimen showed that it was partially transformed from an ideal bond specimen to a good bond specimen after tensile impact. However, it is close to the [Al/0°/90°/0°/Al] sandwich specimen, as the latter specimen experiences a partially transient zone between good bonding and weak bonding, representing higher reduction values of both flexural peak load by 45.8% and deflection (corresponding to peak load) by 15.04% than the [Al/0°/90°/0°/Al] hand lay-up specimen. In addition, [0°/90°/0°] hand lay-up specimen displays the least resistance since it lacks the aluminum plates to absorb the deformation energy, showing the highest reduction in flexural peak load and stiffness values. Thus, the most common damage is intra-damage, such as fiber breakage and matrix cracking.

**Table 8:** The effect of the lay-up technique on the relative damage measured through the flexural test.

Specimen	Relative peak load (%)	Relative deflection corresponding to peak load (%)	Relative max. deflection (%)	Relative flexural stiffness (%)
[Al/0°/90°/0°/Al] hand lay-up	− 22.33	30.65	1.65	− 40.6
[Al/0°/90°/0°/Al] sandwich	− 45.89	− 15.04	− 5.27	− 36.31
[0°/90°/0°] hand lay-up	− 52.21	26.59	26	− 62.25

### **Group 2: effect of inserting [90**°**] layers**

#### Axial tensile-impact test results

**Fig. 6:**
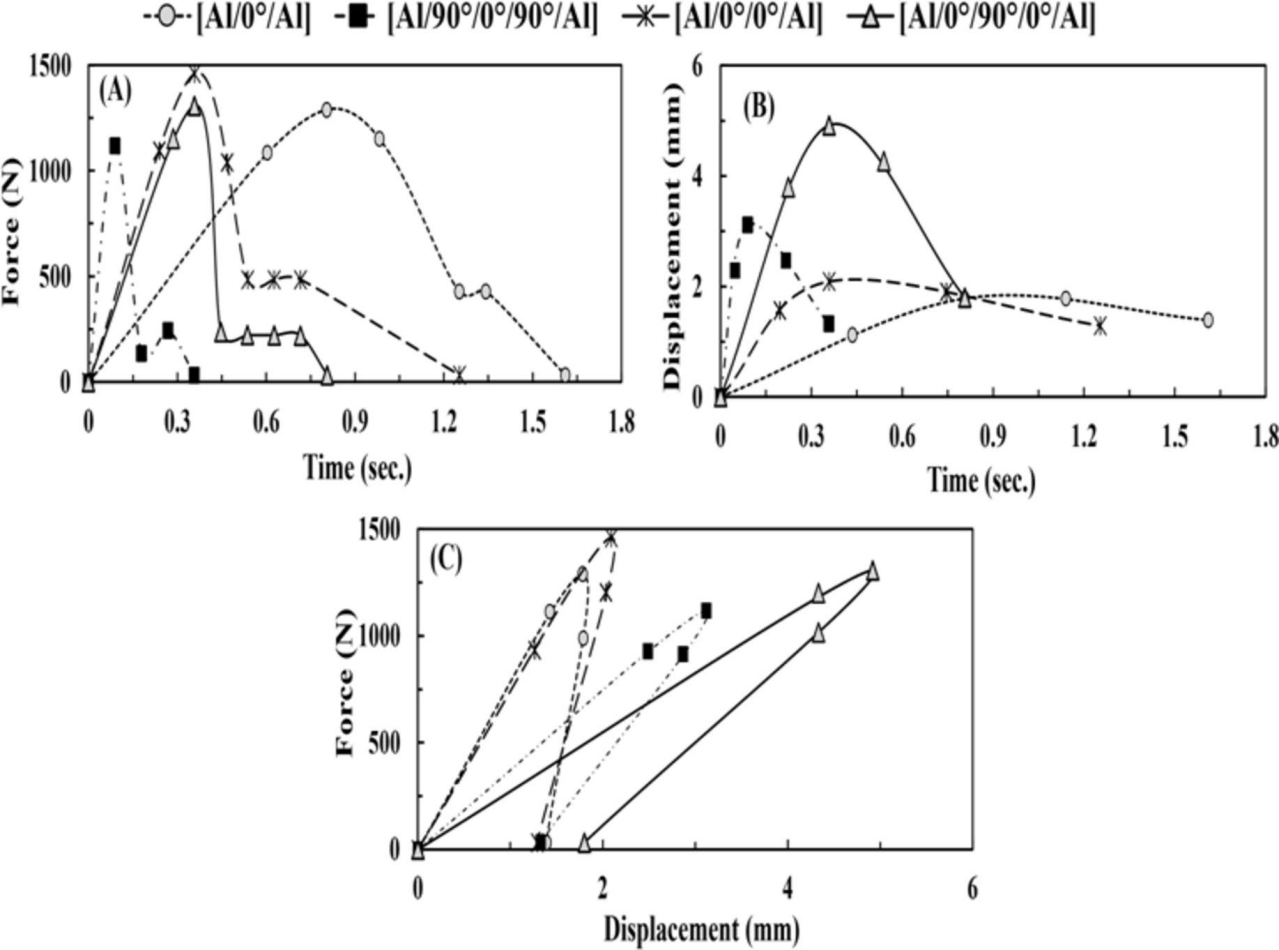
(**A**) Force vs. time (through tensile-impact) for group 2, (**B**) displacement vs. time (through tensile-impact) for group 2, and (**C**) force vs. displacement (through tensile-impact) for group 2.

Table 9; Fig. 6 reveal that the [Al/0°/90°/0°/Al] FML specimen illustrated a greater absorbed energy value of 1.13 *J* more than the [Al/0°/0°/Al] FML specimen, which was assessed by a maximum permanent extension of 1.8 *mm*. This may be because it has three layers of FRP (single middle layer [90°]: acts to break up the crack propagating in the direction of the load, double outer layers [0°]: takes the main effect to absorb the impact energy). Lee et al.^38^ found that the cracks spread more straightforwardly in the unidirectional FRP, while the cross-ply FRP in FMLs could avoid crack growth.

The [Al/0°/90°/0°/Al] benefits from energy absorption from the layers of [0°]. However, compared to [Al/0°/0°/Al] FML specimen, the [Al/0°/90°/0°/Al]’s reduction (in peak load) was due to the intralaminar damage process in the [90°] layer. This is opposed to the [Al/90°/0°/90°/Al] FML specimen, which had a lower absorbed energy than the [Al/0°/Al] FML specimen. Furthermore, it received more interlaminar damage between the FRP layers and aluminum plates. These results agree with those acquired by Cheng et al.^39^, as the [0°] layers in FML specimens possessed higher absorbed energy than those with [90°] layers.

**Table 9:** The experimental results of the tensile-impact test based on the [90°] layers insertion effect.

Specimen	Absorbed energy, (J)	Avg. peak load, (kN)	Permanent extension, (mm)	Standard deviation (σ), (J)	Coefficient of variation (COV%)
[Al/0°/Al]	0.89	1.28	1.39	0.19	14.82
[Al/90°/0°/90°/Al]	0.71	1.12	1.325	0.08	7.06
[Al/0°/0°/Al]	0.93	1.45	1.29	0.02	1.64
[Al/0°/90°/0°/Al]	1.13	1.3	1.8	0.06	4.87

#### Quasi-static tensile test results

**Fig. 7:**
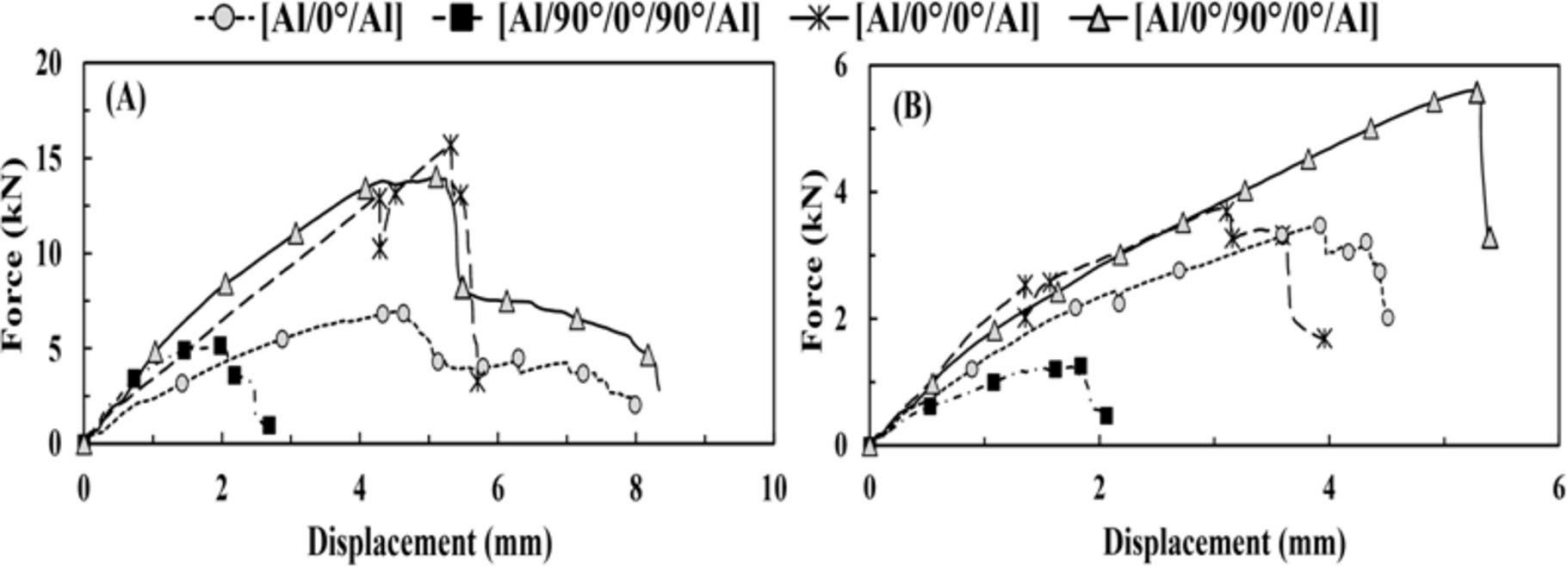
(**A**) Force vs. displacement (without tensile-impact) under tensile test for group 2 and (**B**) force vs. displacement (after tensile-impact) under tensile test for group 2.

It is worth noting that the following comparison is established on the force-displacement curve as an alternative to the stress-strain curve to disregard increasing the FML cross-section specimen because of the insertion of [90°] layers. As depicted in Table 10; Fig. 7(A) for the tensile test, the [Al/0°/0°/Al] FML specimen has the maximum peak load. Despite entering a mid-layer of [90°] as in [Al/0°/90°/0°/Al], its maximum peak load is reduced by 10.5%. Similarly, Hu et al.^40^ and Ergun et al.^41^ found the same findings. Moreover, the [Al/90°/0°/90°/Al] FML specimen includes outermost layers of [90°], which have a devastating effect on keeping a good bond with the aluminum plates, resulting in ruinous delamination in contrast to [Al/0°/Al] FML specimen. Hence, the peak load of [Al/90°/0°/90°/Al] FML specimen is reduced by roughly 27% of the similar specimen without [90°] layers, i.e., [Al/0°/Al] FML specimen.

Conversely, as depicted in Tables 10 and 11; Fig. 7(B) for the TAI test, the [Al/0°/90°/0°/Al] FML specimen has the maximum peak load. However, it showed a lower reduction in tensile peak load by 60.13%, compared to 76.2% for the [Al/0°/0°/Al] FML specimen. The [Al/0°/90°/0°/Al] FML specimen depicted a larger decrease in tensile stiffness by 60.9%, as it received more extra deformation (corresponding to peal load) after TAI test than the [Al/0°/0°/Al] FML specimen. This may be attributed to the following two reasons: the [0°] layers received the most impact damage, while the [90°] layer blunted the generated matrix cracks normal to the load’s direction during the TAI test. Furthermore, the [Al/0°/Al] FML specimen is still outperforming the [Al/90°/0°/90°/Al] FML specimen with less reduction in tensile peak load and tensile stiffness. It is worth noting that the [Al/90°/0°/90°/Al] FML specimen has a devastating effect on the bond strength with the aluminum plates, resulting in severe delamination in contrast to [Al/0°/Al] FML specimen.

The current findings agree with those of Sharma and Velmurugan^42^ for Titanium/-Unidirectional Glass Fiber/epoxy-/Titanium FML specimens. They discovered that the interlaminar damage region increased, following the matrix cracking between [90°] layers and two titanium plates. Moreover, for the [0°] layer under tensile test, the matrix was intact enough to attach to fibers and aluminum well, as it transforms the load for fibers, resulting in fiber breakage. Meanwhile, it restrained aluminum from failing (due to crack propagation) within the fiber-breakage zone. However, for the [0°] layer under the TAI test, the matrix was pre-damaged and could not attach to fibers and aluminum enough, as with fibers. Therefore, matrix crack was easily gained (parallel to the load’s direction) with no fiber breakage. There was no restriction to fail (due to crack propagation) within a certain zone for aluminum.

**Table 10:** The effect of the [90°] layers insertion on the tensile test results.

Specimen	Avg. peak load (kN)		Coefficient of variation (COV%)		Displacement corresponding to peak load (mm)		Max. displacement (mm)	
	Without impact	After impact	Without impact	After impact	Without impact	After impact	Without impact	After impact
[Al/0°/Al]	6.92	3.48	3.2	6.82	4.57	3.92	7.96	4.5
[Al/90°/0°/90°/Al]	5.05	1.24	4.07	7.65	1.92	1.76	2.61	2.068
[Al/0°/0°/Al]	15.67	3.73	1.06	4.03	5.32	3.05	5.71	3.94
[Al/0°/90°/0°/Al]	14.02	5.59	8.23	4.27	5.13	5.24	8.33	5.39

**Table 11:** Effect of the [90°] layers insertion on the relative damage measured through the tensile test.

Specimen	Relative peak load (%)	Relative displacement corresponding to peak load (%)	Relative max. displacement (%)	Relative tensile stiffness (%)
[Al/0°/Al]	− 49.71	− 14.22	− 43.5	− 41.4
[Al/90°/0°/90°/Al]	− 75.45	− 8.33	− 20.8	− 73.21
[Al/0°/0°/Al]	− 76.2	− 42.14	− 30.9	− 58.5
[Al/0°/90°/0°/Al]	− 60.13	2.14	− 35.3	− 60.9

#### Quasi-static flexural test results

**Fig. 8:**
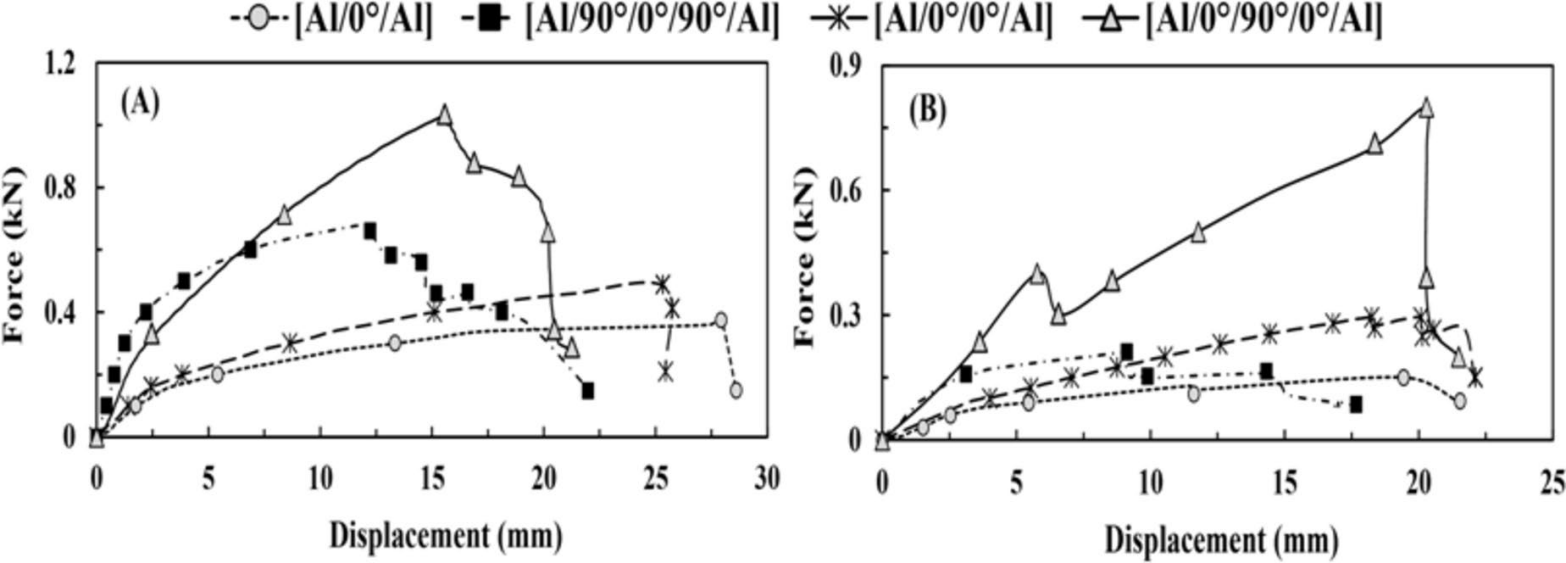
(**A**) Force vs. displacement (without tensile-impact) under flexure test for group 2 and (**B**) force vs. displacement (after tensile-impact) under flexure test for group 2.

In the case of flexural and FAI tests, as shown in Tables 12 and 13; Fig. 8, the [Al/0°/90°/0°/Al] FML specimen demonstrated a higher peak load value and an increase of deflection (corresponding to peak load) by 30.7%. It showed a lower flexural peak load and stiffness by 22.33% and 40.6%, respectively, compared to 37.5% and 12.5% for [Al/0°/0°/Al] FML specimens. However, Hu et al.^14^ and Liu et al.^43^ found that the intermediate [90°] layer does not affect the flexural performance.

The peak load value of peak load for [Al/0°/Al] FML specimen was lower than that of [Al/90°/0°/90°/Al] FML specimen. The [Al/0°/Al] FML specimen showed a greater reduction in the ultimate load (60.53%) due to being subjected to impact load than that reduction in [Al/90°/0°/90°/Al] FML specimen by (69.53%), as listed in Table 13. Furthermore, it showed a greater maximum deflection reduction value of 24.72%. It is worth noting that the delamination caused within the [Al/90°/0°/90°/Al] FML specimen between the two [90°] layers and aluminum plates is not as severe as the tensile test. Also, Li et al.^15^ proved that the failure of the [90°] layer does not affect the delamination. On the other hand, the specimen showed no separation, only distortion and different modes of failures: matrix tension crack (in the [90°] layer), fiber breakage (in the [0°] layer), and delamination between the core and FML’s skin. The two modes of failures, matrix tension crack and fiber breakage, were localized and occurred only at the maximum deflection zone.

**Table 12:** The effect of the [90°] layers insertion on the flexural test results.

Specimen	Avg. peak load (kN)		Coefficient of variation (COV%)		Deflection corresponding to peak load (mm)		Max. deflection (mm)	
	Without impact	After impact	Without impact	After impact	Without impact	After impact	Without impact	After impact
[Al/0°/Al]	0.38	0.15	4.16	5.27	27.9	19.43	28.6	21.53
[Al/90°/0°/90°/Al]	0.68	0.21	6.98	7.53	11.97	9.01	22.16	17.67
[Al/0°/0°/Al]	0.48	0.3	6.59	5.27	25.43	18.16	25.45	22.13
[Al/0°/90°/0°/Al]	1.03	0.8	5.37	3.95	15.53	20.29	21.15	21.5

**Table 13:** Effect of the [90°] layers insertion on the relative damage measured through the flexural test.

Specimen	Relative peak load (%)	Relative deflection corresponding to peak load (%)	Relative max. deflection (%)	Relative flexural stiffness (%)
[Al/0°/Al]	− 60.53	− 30.4	− 24.72	− 43.31
[Al/90°/0°/90°/Al]	− 69.12	− 24.73	− 20.3	− 58.9
[Al/0°/0°/Al]	− 37.5	− 28.6	− 13	− 12.5
[Al/0°/90°/0°/Al]	− 22.33	30.7	1.65	− 40.6

### Progressive damage

This section describes the prevalent damage in each part of the FMLs specimen, i.e., [90°] layers, [0°] layers, and aluminum plates, due to being subjected to tensile impact load. Besides, those under quasi-static tension or flexural loading without or after impact.

#### Axial tensile-impact loading

**Fig. 9:**
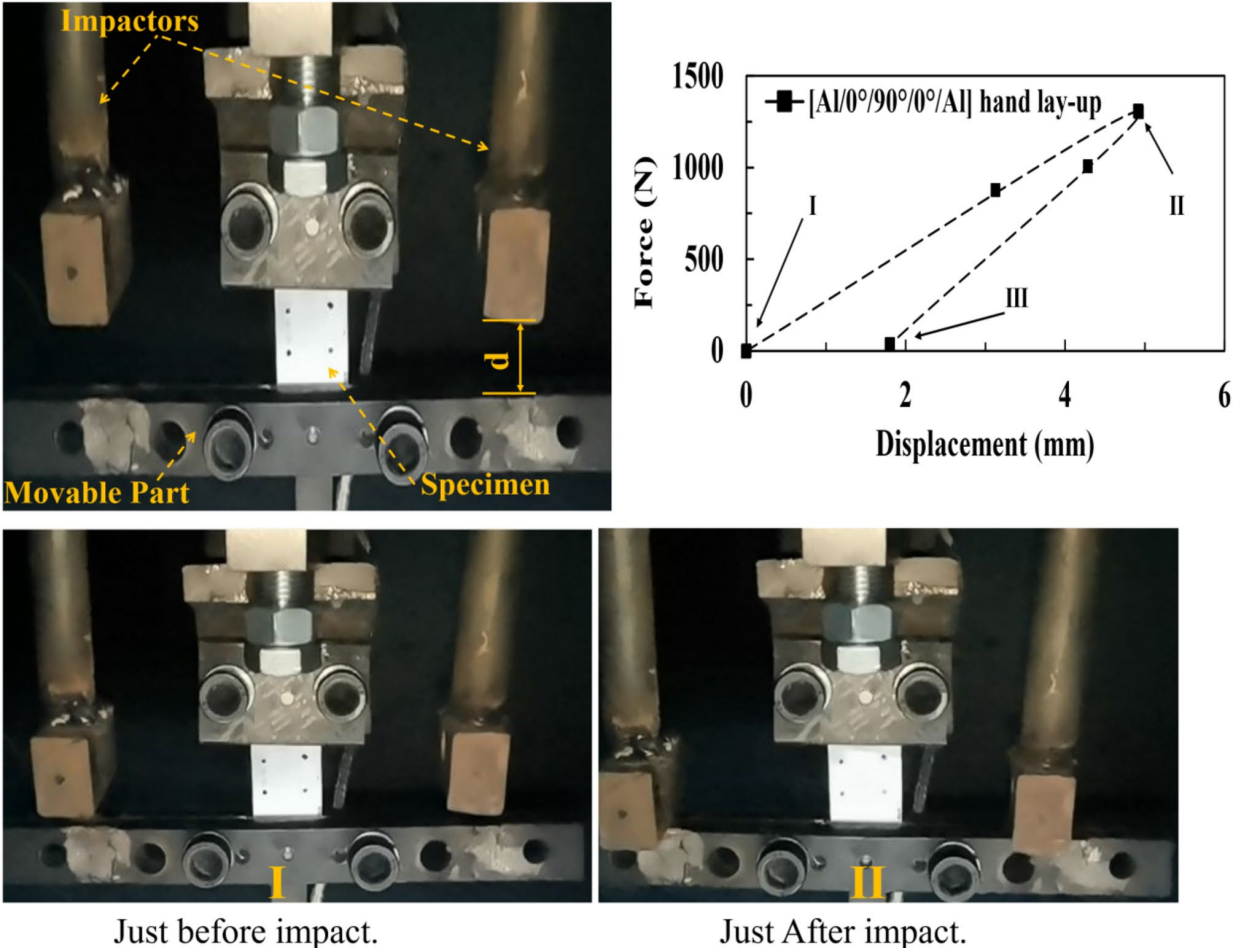
The tensile-impact test stages.

Figure 9 depicts the three critical time loading points in the tensile impact test for the [Al/0°/90°/0°/Al] hand lay-up specimen. It is clear that the beginning of the application of impact load, i.e., Point I (Origin point of the force-displacement curve, 0 *kN*, 0 *mm*, 0 *sec*), occurred when the impactor just before striking the movable part (anvil). At the second point (at point II: maximum impact load), the impactor had reached its maximum distance before rebounding, i.e., 1.3 *kN*, 4.92 *mm*, 0.358 *sec.* At the third point, just at the complete separation between the impactor and the anvil, i.e., the elastic deformation was completely recovered while maintaining the irreversible/permanent deformation in the specimen, 0 *kN*, 1.8 *mm*, 0.806 *sec*. After assessing the third point, the specimens absorbed the low-impact energy, providing two stages of damage: partial delamination between the aluminum plates and FRP and plastic damage in plates. Furthermore, Azhdari et al.^44^ showed that the lowest impact energy had matrix damage, delamination, and plastic deformation, while fiber breakage was detected at higher energy values.

**Fig. 10:**
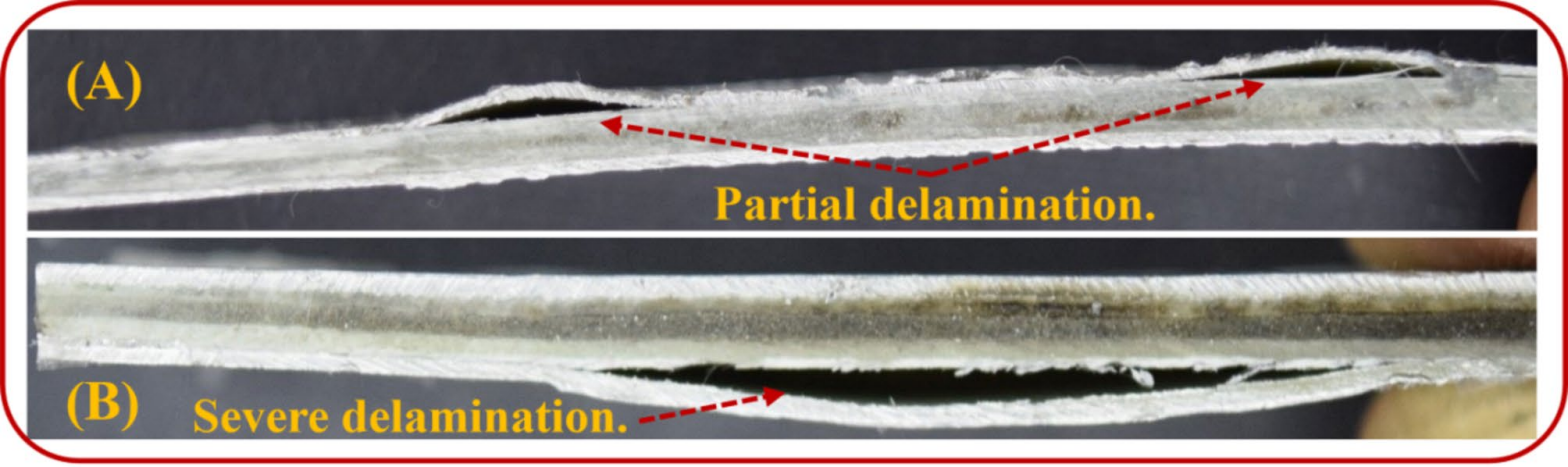
(**A**) The aluminum layer aside from [0°] layer and (**B**) The aluminum layer aside from [90°] layer after tensile-impact.

The unidirectional fiber/epoxy core inside aluminum plates clarified two divisions of delamination: partial delamination aside from [0°] and severe delamination aside from [90°], as shown in Fig. 10. In the case of the [0°] layer, the fiber and matrix suffered from the impact load. However, in the case of the [90°] layer, the matrix carried most of the impact load, showing a tendency to interlaminar damage as delamination.

#### Quasi-static tensile loading

**Fig. 11:**
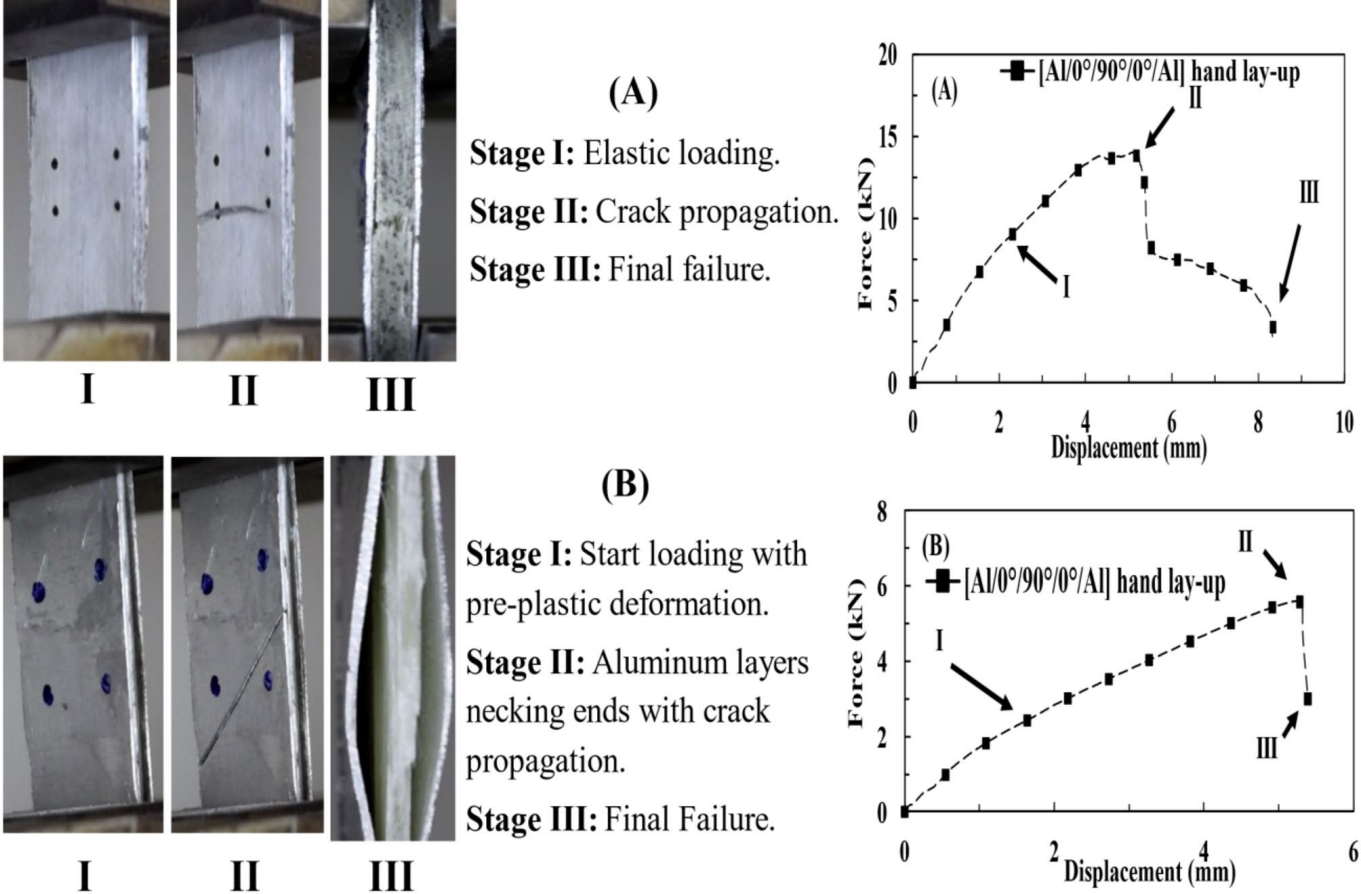
Failure stages of (**A**) the tensile test specimen and (**B**) the TAI test specimen.

The experimental progressive damages under the tensile test without and after tensile impact loading are shown in Fig. 11. The damage of [Al/0°/90°/0°/Al] hand lay-up sample under tensile load without impact indicated three main stages, as shown in Fig. 11(A). The specimens exhibited elastic behavior, i.e., stage (I) with (9.04 *kN*, 2.31 *mm*). They sustained the applied load until the crack initiation when they reached their maximum load, i.e., in stage (II) with (14.02 *kN*, 5.12 *mm*). Further, the cracks grew in the plastic medium (for the core and the metal). Finally, the specimen showed FRP splitting and minor delamination around the failure zone (at the end of the test), i.e., stage (III) with (2.82 *kN*, 8.33 *mm*). Figure 11(B) illustrates the progressive damage under the TAI test. Following aluminum necking at the point of (2.424 *kN*, 1.638 *mm*), stage (I). In stage (II), an inclined crack rapidly appeared across the specimen surface after minor delamination at 5.59 *kN* and 5.243 *mm*. Finally, delamination propagated to a severe one at 3.135 *kN* and 5.385 *mm*, stage (III). The pre-damaged matrix was the main reason for distinguishing between minor delamination (the matrix was still intact) and major delamination (the matrix was pre-impact). It also represented the difference between the crack inclination in the two tests; the more intact the matrix, the less the crack inclination and interlaminar damage.

**Fig. 12:**
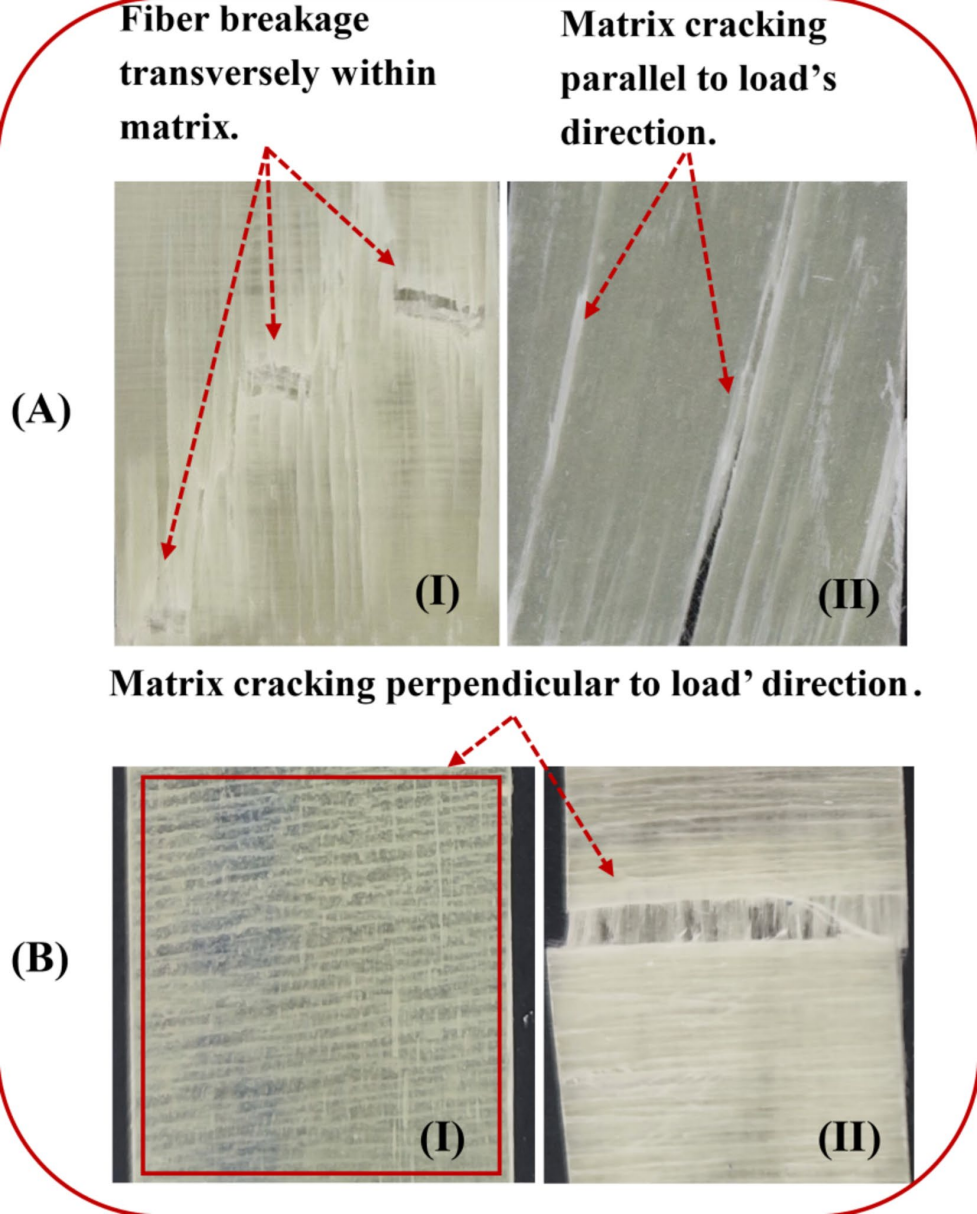
The final failure as: (**A**-I) The [0°] layer after the tensile test, (**A**-II)The[0°] layer after the TAI test, and (**B**-I & II)The[90°] layer after the tensile and TAI tests.

The final failure of the unidirectional fiber/epoxy core is shown in Fig. 12(A - I). The [0°] fiber breakage (transversely within the matrix) was demonstrated through the tensile test, as the matrix transferred the load uniformly to fibers, and the fibers could endure the load till breakage. Furthermore, an aggressive matrix cracking, parallel to the load’s direction, was detected through TAI, as the matrix transferred part of the load to the fibers due to the pre-damaged matrix. The matrix failed before transferring most of the tensile load to fibers, as shown in Fig. 12(A-II). Additionally, the [90°] layer exhibited a matrix crack, which appeared at both tensile and TAI tests. Because the fibers were normal to the load, they lost their effectiveness (as it is very effective when parallel to load), as depicted in Fig. 12 (B-I & II).

**Fig. 13:**
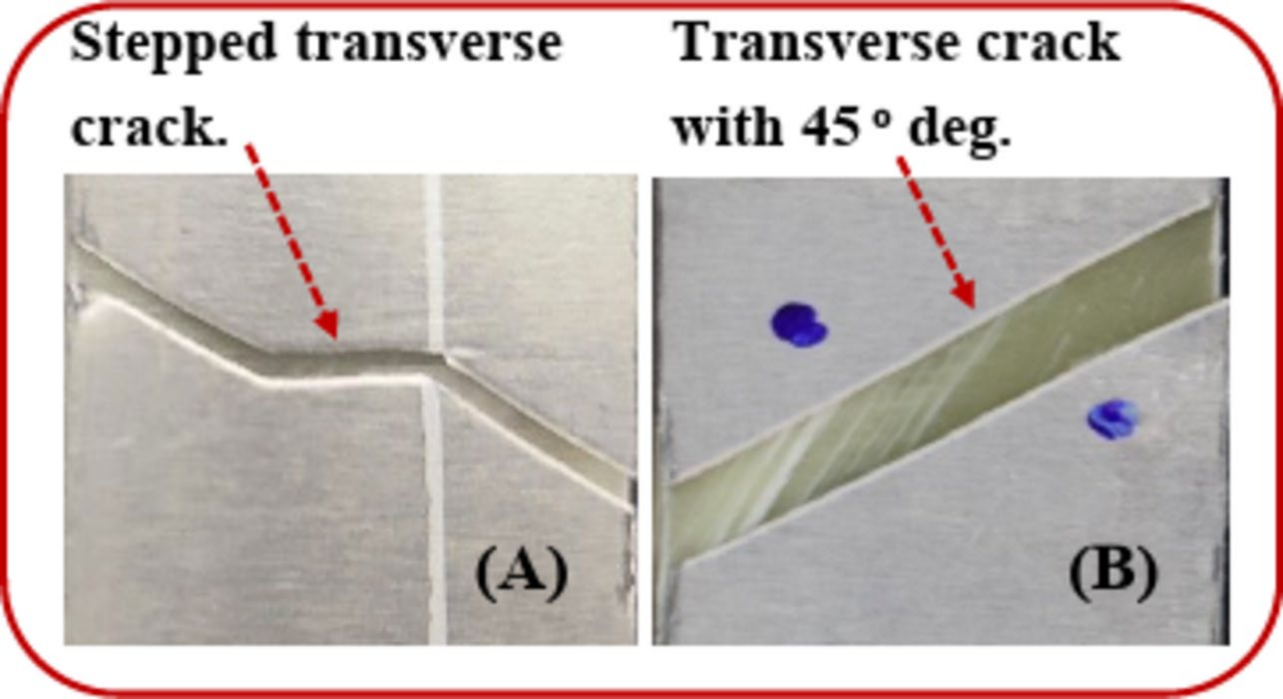
The final failure: (**A**) the aluminum layer after the tensile test and (**B**) the aluminum layer after the TAI test.

Figure 13(A) shows the transversely stepped crack passed through aluminum plates under the tensile test (without tensile impact). Because the matrix endured the load, it retarded the crack propagation. In the case of TAI, the aluminum plate showed plastic damage at 45° and an inclined crack through the aluminum, as shown in Fig. 13(B). The path of the crack passed through almost with no resistance from the matrix (due to the pre-damaged matrix crack). A very located delamination occurred as the last event (around the crack between aluminum and unidirectional fiber/epoxy). Likewise, Kumar et al.^45^ found under tensile loading that the failure of [Al/CFRP/Al/CFRP/Al] (CARALL) laminates into three steps, as in step I, elastic deformation was noticed in the aluminum and CFRP. In step II, elastic deformation was noticed in CFRP, but plastic deformation in aluminum. Suddenly, a load drop in step III was assigned to interlaminar or intralaminar damage^45^. Likewise, for the study of the residual tensile behavior, the failure modes were observed by Yao et al.^31^ as aluminum cracking, fiber breakage, and delamination between aluminum plates and composite laminates, which were detected around the impact region.

#### Quasi-static flexural loading

**Fig. 14:**
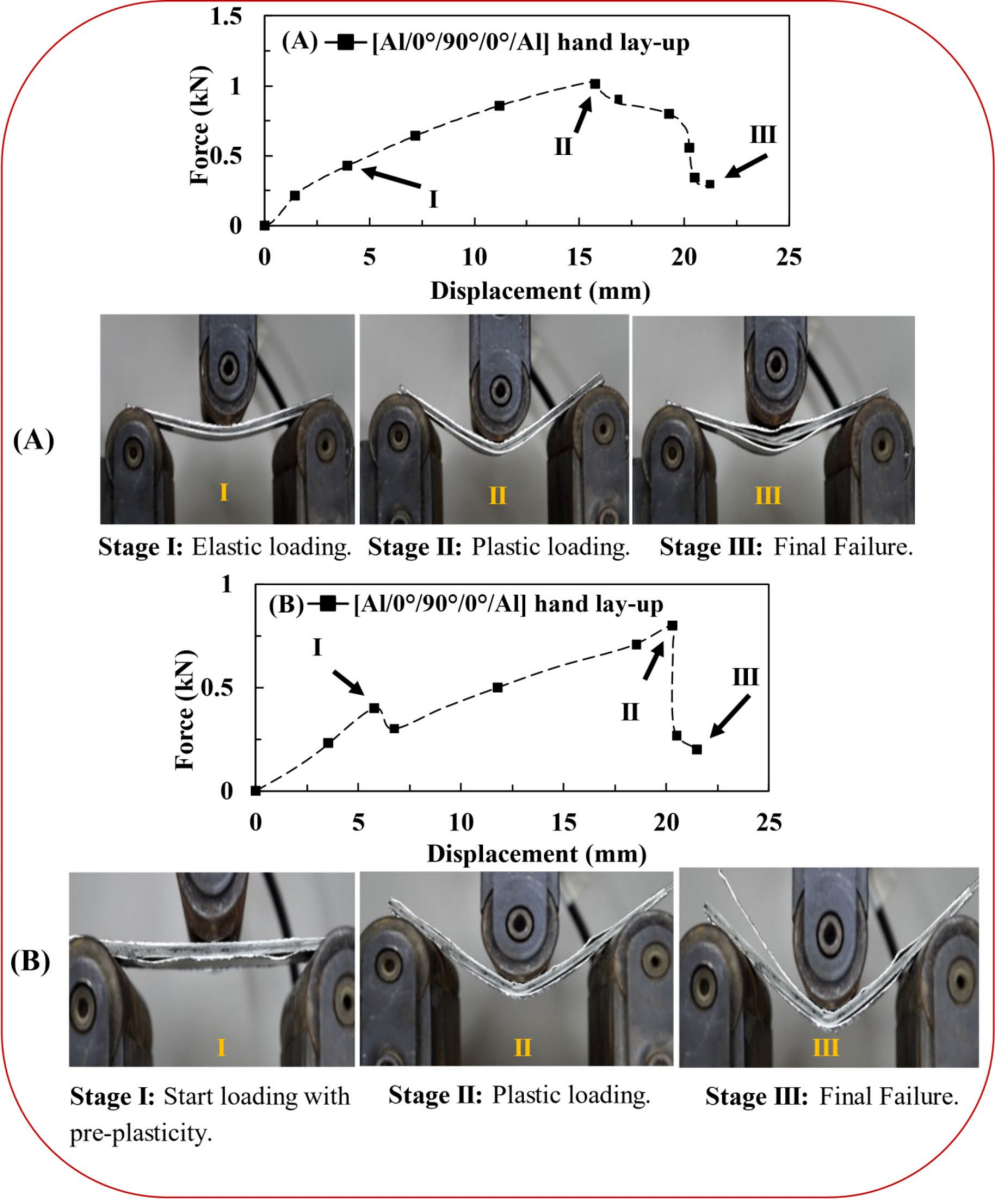
(**A**) The flexural test stages and (**B**) the FAI test stages.

Figure 14 shows the progressive damage of the [Al/0°/90°/0°/Al] hand lay-up specimen under flexural tests without/after tensile impact. There are three major stages of damage through flexural tests (without/after tensile impact). At the beginning of loading, the specimens exhibit elastic behavior, i.e., stage (I): 0.43 *kN* and 3.94*mm* for the flexural test and 0.4 *kN* and 5.77 *mm* for the FAI test. Then, the plastic flow appeared, where all the specimens achieved the peak load, stage (II): 1.03*kN* and 15.53 *mm* for the flexural test and 0.8*kN* and 20.29 *mm* for the FAI test. The fiber, matrix, and aluminum were subjected to damage modes, especially the matrix detachment between FRP and aluminum. Moreover, the FAI test specimen seemed to have a more catastrophic plastic failure (large-scale delamination) than the corresponding one conducted without tensile impact, stage (III): 0.27 *kN* and 21.15 *mm* for the flexural test and 0.2 *kN* and 21.49 *mm* for the FAI test. It is worth noting that the matrix in the latter case still provided endurance to the bending load (as the matrix started as an intact matrix).

**Fig. 15:**
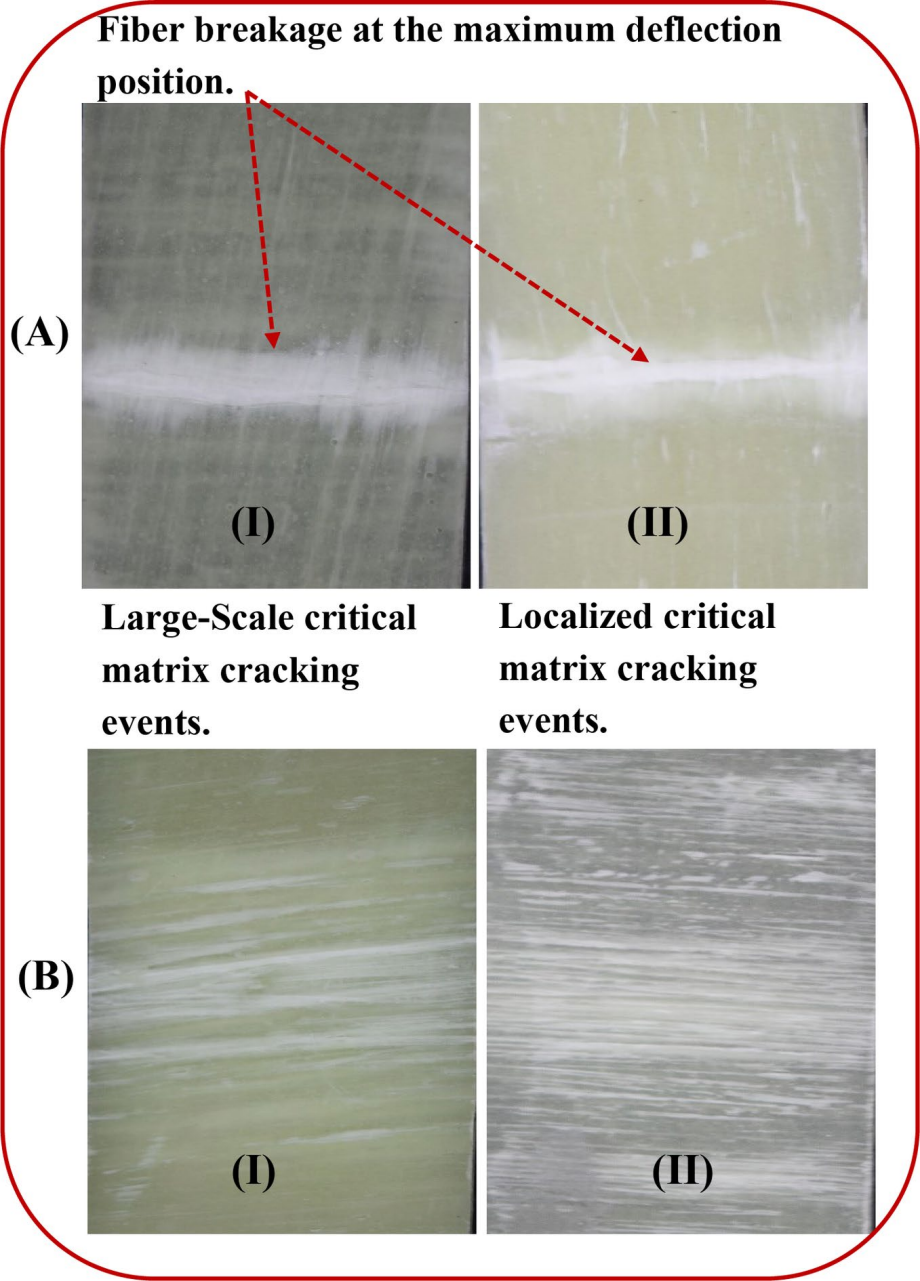
The final failure as: (**A**-I & II) The [0°] layer after the flexural & FAI tests, (**B**-I) The [90°] layer after the flexural test, and (**B**-II)The[90°] layer after the FAI tests.

Figure 15 (A) further illustrates the final failure of the [0°] layer due to flexural and FAI loadings. It is clear that the [0°] layer depicts fiber-kinking transversely within the matrix for both cases in the same manner. Additionally, the [90°] layer for the unidirectional fiber/epoxy core exhibited matrix cracking parallel to the direction of the fibers, as illustrated in Fig. 15(B). Notably, many critical matrix cracks occurred in specimens following tensile impact, while less critical ones occurred in specimens without tensile impact. This may be due to the matrix in the latter case started as an intact matrix. It is worth noting that the two damage modes (fiber-kinking and matrix crack) happened in the maximum deflection location.

**Fig. 16:**
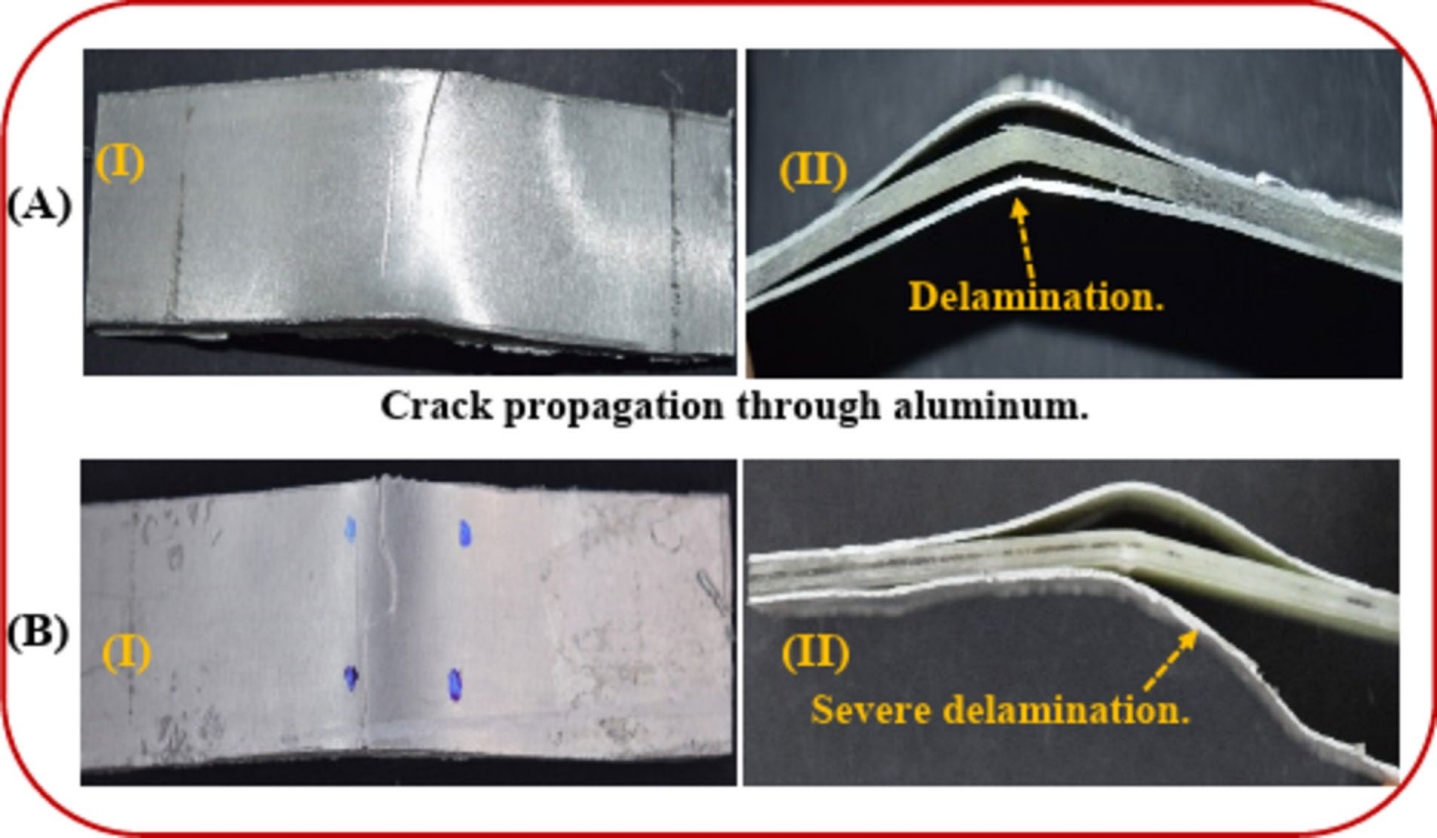
The final failure: (**A**) the aluminum layer after the flexural test and (**B**) the aluminum layer after the FAI test.

Moreover, in the case of aluminum plates under the flexural test without impact, a partial transverse crack (in the metal) was produced during the delamination process between the core and aluminum shield, as shown in Fig. 16(A). In the case of the FAI test, the aluminum plates indicate plastic damage ended with a transversely wide crack through the aluminum, as shown in Fig. 16(B). However, it is obvious that the FAI test illustrated an increase in the degradation of the aluminum, through which the crack can initiate and propagate. Also, the severe delamination process between the composite core and aluminum plates is presented. As Azhdari et al.^44^ mentioned, delamination is prevalent in lower-impact energy.

## Conclusions

After evaluating the behavior of FML specimens through TAI and FAI tests compared to the corresponding virgin specimens, the following conclusions can be drawn:

- The [Al/0°/90°/0°/Al] sandwich specimen had the strongest resistance to external force for the tensile, flexural, and TAI tests, compared to [Al/0°/90°/0°/Al] and [0°/90°/0°] hand lay-up specimens.
- The sandwich technique provided the best choice of the lay-up technique, as it presented a good bond between aluminum and FRP. Therefore, energy was consumed into interlaminar damage. However, the hand lay-up lost this advantage due to its perfect bond between the aluminum and FRP, i.e., it behaved as a monolithic material.
- For the TAI test, the [Al/0°/90°/0°/Al] FML specimen had a maximum peak load more than the [Al/0°/0°/Al] FML specimen. For the flexural and FAI tests, the [Al/0°/90°/0°/Al] FML specimen demonstrated a higher peak load value than [Al/0°/0°/Al] FML specimens.
- The insertion of the [90°] as a mid-layer increased the ability to endure the damage, especially when the damage was created due to tensile-impact loading. However, inserting the [90°] as an outer layer of the core decreased the ability to bear the damage, making a critical delamination.
- The main failure modes shown in the tensile, flexural, TAI, and FAI were fiber breakage (in the [0°] layer), matrix cracking (in the [0°] and [90°] layers), and delamination (between the FRP and aluminum).

## Electronic supplementary material

Below is the link to the electronic supplementary material.

Supplementary Material 1

Supplementary Material 2

## Data Availability

The authors declare that the data supporting the findings of this study are available within the paper.
