# Supplementary material for: Quasistatic tensile and flexural behaviors of fiber metal laminates after subjecting to uniaxial tensile impact loading
Source: Sci Rep. 2025 May 8;15:16051. doi: 10.1038/s41598-025-99159-6 (PMC12062328; doi:10.1038/s41598-025-99159-6)
Supplement: Supplementary file 1 — Supplementary Material 1 [file 41598_2025_99159_MOESM1_ESM.docx]

Table S1 showed a statistical (repeatability) analysis: experimental standard deviation and coefficient of variation(relative standard deviation), COV, for the tensile, flexural, and tensile impact tests. The values of the standard deviation and COV of the present results showed the accuracy, reliability, and repeatability of all measurements as follows:

- For the tensile test (without tensile impact), the COV ranged from 1.06% to 8.23% for the peak load and from 1.22% to 7.23% for the displacement measurements. In the case of peak load, the stacking sequences without 90° ply ([Al/0°/Al] and [Al/0°/0°/Al]) showed the lowest values of COV. All stacking sequences had a COV of maximum displacement greater than that of displacement at peak load except ([Al/0°/Al]).
- For the tensile test (after tensile impact), the COV ranged from 2.73% to 7.65% for the peak load and from 1.1% to 8.19% for the displacement measurements. The sandwich lay-up technique showed lower COVs of the peak load and its corresponding displacement compared to the hand lay-up technique, showing more reliability. However, the hand lay-up technique showed a lower COV of maximum displacement.
- For the flexural test (without tensile impact), the COV ranged from 4.16% to 17.87% for the peak load and from 0.36% to 1.52% for the displacement measurements. All stacking sequences showed lower COVs of the displacement measurements compared to the COVs of the peak load. All stacking sequences depicted higher COVs of the maximum displacement, compared to that corresponding to the peak load, except ([Al/90°/0°/90°/Al] and [Al/0°/90°/0°/Al] by hand lay-up). It is worth noting that the [Al/0°/Al] specimens had the lowest COVs of the peak load and its corresponding displacement, while the [Al/90°/0°/90°/Al] specimens possessed the lowest COV of the maximum displacement.
- For the flexural test (after tensile impact), the COV ranged from 3.95% to 12.01% for the peak load and from 0.64% to 2.83% for the displacement measurements. As is already known, the flexural test had complex loading of the tension on the upper side of the specimen and the compression on the lower side of the specimen, indicating that for both flexural tests (without and after tensile impact), the samples presented lower the COVs of the displacement measurements, compared to the COVs of the peak load. It is worth noting that the [Al/0°/90°/0°/Al] by hand lay-up specimens received the lowest COVs of the peak load and its corresponding displacement, while the [Al/0°/90°/0°/Al] by sandwich lay-up specimens owned the lowest COV of the maximum displacement.
- For the tensile impact test, the COV ranged from 2.42% to 8.97% for the peak load, 0.75% to 3.34% for the permanent extension, and 1.64% to 18.65% for the absorbed energy. All stacking sequences displayed lower COVs of the permanent extension than that of the peak load and the absorbed energy, except the [Al/0°/0°/Al], which had a lower COV of the absorbed energy than that of the peak load and the permanent extension. The [0°/90°/0°] specimens showed the lowest COV of the peak load, while the [Al/0°/0°/Al] specimens showed the highest COV of the peak load.
- In summary, regarding the minimum and maximum COVs for all tests, the tensile test (without tensile impact) showed the lowest COV of the peak load by the [Al/0°/0°/Al] specimens. Meanwhile, the flexural test (without tensile impact) showed the highest COV of the peak load by the [Al/0°/90°/0°/Al] by sandwich lay-up technique. The flexural test (without tensile impact) presented the lowest COV of the displacement corresponding to the peak load by the [Al/0°/Al] specimens, while the tensile test (after tensile impact) exhibited the highest COV of the displacement corresponding to the peak load by the [Al/90°/0°/90°/Al] specimens. The flexural test (after tensile impact) depicted the lowest COV of the maximum displacement by the [Al/0°/90°/0°/Al] by sandwich lay-up specimens, as the tensile test (without tensile impact)exposed the highest COV of the maximum displacement by the [0°/90°/0°] specimens. The tensile impact test illustrated the lowest COV of the absorbed energy by the [Al/0°/0°/Al] specimens. Moreover, it showed the highest COV of the absorbed energy by the [Al/0°/90°/0°/Al] by sandwich lay-up specimens. The tensile impact test displayed the lowest COV of the permanent extension by the [Al/90°/0°/90°/Al]specimens. Also, it showed the highest COV of the permanent extension by the [Al/0°/90°/0°/Al] by sandwich lay-up specimens.
