# Supplementary material for: Quasistatic tensile and flexural behaviors of fiber metal laminates after subjecting to uniaxial tensile impact loading
Source: Sci Rep. 2025 May 8;15:16051. doi: 10.1038/s41598-025-99159-6 (PMC12062328; doi:10.1038/s41598-025-99159-6)
Supplement: Supplementary file 2 — Supplementary Material 2 [file 41598_2025_99159_MOESM2_ESM.docx]

**Table S1** The experimental standard deviations (**σ**) and coefficients of variation (**COV**) for the tensile, flexural, and tensile-impact tests.

| **Specimen** | **Avg. Peak Load (*kN*)** | **σ  (*kN*)** | **COV  (*%*)** | **Disp. at Peak Load  (*mm*)** | **σ  (*mm*)** | **COV  (*%*)** | **Max. Disp.  (*mm*)** | **σ  (*mm*)** | **COV  (*%*)** |
| --- | --- | --- | --- | --- | --- | --- | --- | --- | --- |
| **Tensile Test (Without Tensile-Impact)** | | | | | | | | | |
| **[Al/0°/Al]  hand lay-up** | **6.92** | **0.22** | **3.2** | **4.57** | **0.13** | **2.85** | **7.96** | **0.122** | **1.53** |
| **[Al/0°/0°/Al] hand lay-up** | **15.67** | **0.17** | **1.06** | **5.32** | **0.14** | **2.54** | **5.71** | **0.29** | **5.17** |
| **[Al/0°/90°/0°/Al]  sandwich lay-up** | **17.69** | **0.64** | **3.62** | **5.73** | **0.09** | **1.59** | **6.84** | **0.24** | **3.54** |
| **[Al/0°/90°/0°/Al]  hand lay-up** | **14.02** | **1.15** | **8.23** | **5.13** | **0.062** | **1.22** | **8.33** | **0.2** | **2.43** |
| **[Al/90°/0°/90°/Al]  hand lay-up** | **5.05** | **0.21** | **4.07** | **1.92** | **0.04** | **1.88** | **2.61** | **0.19** | **7.12** |
| **[0°/90°/0°]  hand lay-up** | **6.68** | **0.25** | **3.79** | **2.24** | **0.13** | **6.2** | **2.78** | **0.2** | **7.23** |
|  |  |  |  |  |  |  |  |  |  |
| **Tensile Test (After Tensile-Impact)** | | | | | | | | | |
| **[Al/0°/Al]  hand lay-up** | **3.48** | **0.24** | **6.82** | **3.92** | **0.12** | **3.05** | **4.5** | **0.3** | **6.67** |
| **[Al/0°/0°/Al] hand lay-up** | **3.73** | **0.15** | **4.03** | **3.05** | **0.05** | **1.5** | **3.94** | **0.144** | **3.7** |
| **[Al/0°/90°/0°/Al]  sandwich lay-up** | **6.65** | **0.18** | **2.73** | **6.14** | **0.23** | **3.67** | **7.82** | **0.23** | **2.9** |
| **[Al/0°/90°/0°/Al]  hand lay-up** | **5.59** | **0.09** | **4.27** | **5.24** | **0.11** | **2.04** | **5.39** | **0.1** | **1.86** |
| **[Al/90°/0°/90°/Al]  hand lay-up** | **1.24** | **0.09** | **7.65** | **1.76** | **0.14** | **8.19** | **2.068** | **0.023** | **1.1** |
| **[0°/90°/0°]  hand lay-up** | **3.22** | **0.22** | **6.87** | **3.94** | **0.31** | **7.78** | **4.52** | **0.17** | **3.8** |
|  |  |  |  |  |  |  |  |  |  |
| **Flexural Test (Without Tensile-Impact)** | | | | | | | | | |
|  |  |  |  |  |  |  |  |  |  |
| **[Al/0°/Al]  hand lay-up** | **0.38** | **0.02** | **4.16** | **27.9** | **0.1** | **0.36** | **28.6** | **0.27** | **0.93** |
| **[Al/0°/0°/Al] hand lay-up** | **0.48** | **0.03** | **6.59** | **25.43** | **0.15** | **0.6** | **25.45** | **0.22** | **0.86** |
| **[Al/0°/90°/0°/Al]  sandwich lay-up** | **1.46** | **0.26** | **17.87** | **20.81** | **0.1** | **0.48** | **21.8** | **0.24** | **1.07** |
| **[Al/0°/90°/0°/Al]  hand lay-up** | **1.03** | **0.06** | **5.37** | **15.53** | **0.24** | **1.52** | **21.15** | **0.18** | **0.85** |
| **[Al/90°/0°/90°/Al]  hand lay-up** | **0.68** | **0.05** | **6.98** | **11.97** | **0.11** | **0.92** | **22.16** | **0.15** | **0.69** |
| **[0°/90°/0°]  hand lay-up** | **1.36** | **0.11** | **8.14** | **14.89** | **0.12** | **0.77** | **15.34** | **0.16** | **1.04** |
|  |  |  |  |  |  |  |  |  |  |
| **Flexural Test (After Tensile-Impact)** | | | | | | | | | |
|  |  |  |  |  |  |  |  |  |  |
| **[Al/0°/Al]  hand lay-up** | **0.15** | **0.01** | **5.27** | **19.43** | **0.55** | **2.83** | **21.53** | **0.45** | **2.09** |
| **[Al/0°/0°/Al] hand lay-up** | **0.3** | **0.02** | **5.27** | **18.16** | **0.23** | **1.27** | **22.13** | **0.18** | **0.79** |
| **[Al/0°/90°/0°/Al]  sandwich lay-up** | **0.79** | **0.09** | **12.01** | **17.68** | **0.29** | **1.66** | **20.65** | **0.13** | **0.64** |
| **[Al/0°/90°/0°/Al]  hand lay-up** | **0.8** | **0.03** | **3.95** | **20.29** | **0.14** | **0.7** | **21.5** | **0.31** | **1.44** |
| **[Al/90°/0°/90°/Al]  hand lay-up** | **0.21** | **0.02** | **7.53** | **9.01** | **0.14** | **1.52** | **17.67** | **0.16** | **0.93** |
| **[0°/90°/0°]  hand lay-up** | **0.65** | **0.04** | **6.08** | **18.85** | **0.8** | **0.95** | **19.33** | **0.22** | **1.13** |
|  |  |  |  |  |  |  |  |  |  |
| **Tensile-Impact Test** | | | | | | | | | |
| **Specimen** | **Avg. Peak Load (*kN*)** | **σ  (*kN*)** | **COV  (*%*)** | **Absorbed Energy  (*J*)** | **σ  (*J*)** | **COV  (*%*)** | **Permanent Extension  (*mm*)** | **σ  (*mm*)** | **COV  (*%*)** |
|  |  |  |  |  |  |  |  |  |  |
| **[Al/0°/Al]  hand lay-up** | **1.28** | **0.036** | **2.82** | **0.93** | **0.19** | **14.82** | **1.39** | **0.036** | **2.6** |
|  |  |  |  |  |  |  |  |  |  |
| **[Al/0°/0°/Al] hand lay-up** | **1.45** | **0.13** | **8.97** | **0.89** | **0.02** | **1.64** | **1.29** | **0.03** | **2.33** |
|  |  |  |  |  |  |  |  |  |  |
| **[Al/0°/90°/0°/Al]  sandwich lay-up** | **0.89** | **0.027** | **2.97** | **1.6** | **0.17** | **18.65** | **3.64** | **0.12** | **3.34** |
|  |  |  |  |  |  |  |  |  |  |
| **[Al/0°/90°/0°/Al]  hand lay-up** | **1.3** | **0.1** | **7.69** | **1.13** | **0.06** | **4.87** | **1.8** | **0.045** | **2.54** |
|  |  |  |  |  |  |  |  |  |  |
| **[Al/90°/0°/90°/Al]  hand lay-up** | **1.12** | **0.098** | **8.79** | **0.71** | **0.08** | **7.06** | **1.33** | **0.01** | **0.75** |
|  |  |  |  |  |  |  |  |  |  |
| **[0°/90°/0°]  hand lay-up** | **1.09** | **0.026** | **2.42** | **1.21** | **0.03** | **2.9** | **2.27** | **0.03** | **1.32** |
|  |  |  |  |  |  |  |  |  |  |
